# Can Type of Instrumentation and Activation of the Final Irrigant Improve the Obturation Quality in Oval Root Canals? A Push-Out Bond Strength Study

**DOI:** 10.3390/biology11010059

**Published:** 2022-01-01

**Authors:** Ajinkya M. Pawar, Anda Kfir, Zvi Metzger, Anuj Bhardwaj, Yeyen Yohana, Dian Agustin Wahjuningrun, Alexander Maniangat Luke, Bhaggyashri A. Pawar

**Affiliations:** 1 Department of Conservative Dentistry and Enododntics, Nair Hospital Dental College, Mumbai 400008, India; 2 Department of Endodontology, The Goldschleger School of Dental Medicine, Tel Aviv 69978, Israel; dr.adakfir@gmail.com (A.K.); metzger.zvi@gmail.com (Z.M.); 3 Department of Conservative Dentistry and Endodontics, College of Dental Sciences & Hospital, Indore 453331, India; dranuj_84@yahoo.co.in; 4 Department of Conservative Dentistry, Faculty of Dental Medicine, Universitas Airlingga, Surabaya 60132, East Java, Indonesia; yeyen.yohana-2020@fkg.unair.ac.id; 5 Department of Clinical Science, College of Dentistry, Ajman University, Al-Jurf, Ajman 346, United Arab Emirates; 6 Center of Medical and Bio-allied Health Sciences Research, Ajman University, Al-Jurf, Ajman 346, United Arab Emirates; 7 Division of District Early Intervention Centre, Department of Dentistry, Thane Civil Hospital, Thane 400601, India; bhaggyashripawaar@gmail.com

**Keywords:** AH Plus, C-Points, EndoActivator, EndoSequence bioceramic sealer, instrumentation, oval canals, passive ultrasonic irrigation, push-out bond strength, SAF

## Abstract

**Simple Summary:**

The complete instrumentation of oval root canals remains practically unattainable. The majority of studies show that shaping oval, flat, and irregularly shaped canals is challenging, with more than half of the root canal area remaining unaltered. Furthermore, both rotary and reciprocating files compact hard tissue debris into the isthmus areas and buccal and/or lingual recesses of oval canals, impairing debridement and filling. In addition, one of the most essential variables in assessing the success of endodontic therapy is the adhesion of root canal filling material to dentin.

**Abstract:**

To appraise the outcome of file systems and activation of the final irrigant on the push-out bond strength of root fillings in oval canals. Single-rooted mandibular premolars (*n* = 180) with oval canals were divided into three groups (*n* = 60) for instrumentation: ProTaper Next (PTN), WaveOne (WO), and Self-adjusting File (SAF). The specimens were further divided into subgroups (*n* = 20) and subjected to final irrigation with activation by EndoActivator or passive ultrasonic irrigation or without activation. Then, the specimens were again subdivided (*n* = 10) and obturated with gutta-percha and AH Plus (GP-AH) or C-Point with EndoSequence bioceramic sealer (C-EBC). One-millimeter-thick horizontal slices were cut from the apical third of the root, 5 mm from the apex, and subjected to push-out bond strength (BS) testing. Specimens for which SAF was used exhibited higher BS values than those for which PTN or WO was used (*p* < 0.05). Activation of the final irrigation did not affect the BS of the root fillings. Root fillings made of C-EBC presented a higher BS than those made of GP-AH (*p* < 0.05). Adhesive failure was more common with specimens instrumented using PTN and WO. Root canals instrumented with SAF, showed the highest bond strength values for both root filling materials. The C-EBC produced significantly higher bond strength values than those of the GP-AH.

## 1. Introduction

Clinical endodontics is flooded with an abundance of innovations in terms of instrument design, metallurgy, and kinematics [1]. Most file systems can productively clean canals with a round cross section. Nevertheless, effective cleaning of oval canals remains a challenge [2]. Furthermore, the use of rotary or reciprocating file systems may actively push and accumulate debris in the uninstrumented anatomical eccentricities of root canal systems [2,3,4]. It has been suggested that irrigant activation with either sonic or ultrasonic devices may overcome this problem and remove any tissue or debris remaining in areas untouched by the rotating file [5]. Such remaining debris may further impede the goal of a well-filled and well-sealed root canal system [4].

A previous study has demonstrated that root fillings in oval canals prepared with the Self-adjusting File (SAF) system presented a higher push-out strength than those prepared with a WaveOne file [6]. This was attributed to debris remaining in or packed into uninstrumented canal recesses by the reciprocating file, which prevented the root filling from achieving an intimate contact with the radicular canal wall, thus reducing the push-out strength of the root canal fillings [6]. Nevertheless, in that study no irrigant activation was used.

Irrigation activations improve sealer penetration into the dentinal tubules and improve retention of the filling material [7].

To test whether the root filling material may affect the push-out bond strength after irrigation activation, two root filling methods were used: traditional lateral compaction with gutta-percha and AH-plus sealer, and relatively new C-Points with a bioceramic sealer. Each of them represents a different potential mode of adhesion to the root canal dentin surface. A root canal sealer should adhere to both the core material and the root dentin to preserve the apical seal. Endodontic sealers’ adhesive characteristics may differ given the difference in their chemical components, which may affect their interaction with dentin or core material. Because of its creep capacity and prolonged setting time, AH Plus, an epoxy resin-based sealer, has superior penetration into micro-irregularities, increasing the mechanical interlocking between sealer and root dentin. This fact, together with the cohesiveness of sealer molecules, improves resistance to removal and/or displacement from dentin, which amounts to increased adhesion [8]. Bioceramic sealer adhesion strength, on the other hand, is determined by binding to mineralized dentin tissues. Bioceramic sealers utilize the water found in the dentinal tubules to perform the hydration process that promotes hardening [9].

On the above background, the current study was designed to assess the outcome of different root canal instrumentation (rotary and reciprocating files) and irrigant activation techniques on the push-out strength of two types of root canal fillings in oval canals prepared with the SAF serving as a positive control. The null hypothesis tested was that the activation of the irrigant would overcome the limitations of rotary or reciprocating files in oval canals and increase the push-out strength of the root canal fillings.

## 2. Materials and Methods

### 2.1. Specimen Preparation

The Universitas Airlangaa Faculty of Dental Medicine Health Research Ethical Clearance Commission approved the current study (182/HRECC.FODM/VIII/2021 on 24 August 2021). G-Power 3.1.9.2 was used to determine the sample size. Referring to previous literature [6,10], a power analysis was performed using α = 0.05, power = 0.80, and an effect size of 0.91. A total of 180 sample sizes were determined, with each group having *n* = 60.

In the present study, human mandibular first premolars (*n* = 180) with a single oval root canal were selected from a pool of recently extracted teeth (not older than 90 days) that were extracted for reasons unassociated to the present study. Periapical radiographs were obtained from buccolingual and mesiodistal projections to confirm the presence of a single canal and the shape of the canal. Following confirmation of single canals, the samples were exposed to cone-beam computed tomography scanning (CBCT) to ascertain oval canals. In the axial sections, only teeth with root canals having a buccolingual canal proportions 2.5 times larger than the mesiodistal, at 5 mm from the anatomic apex, were chosen for the present study [11]. The samples were stored in normal saline until use. They were also observed under a stereomicroscope to eliminate any samples with cracks and craze lines.

### 2.2. Stages of the Study

**Figure 1:**
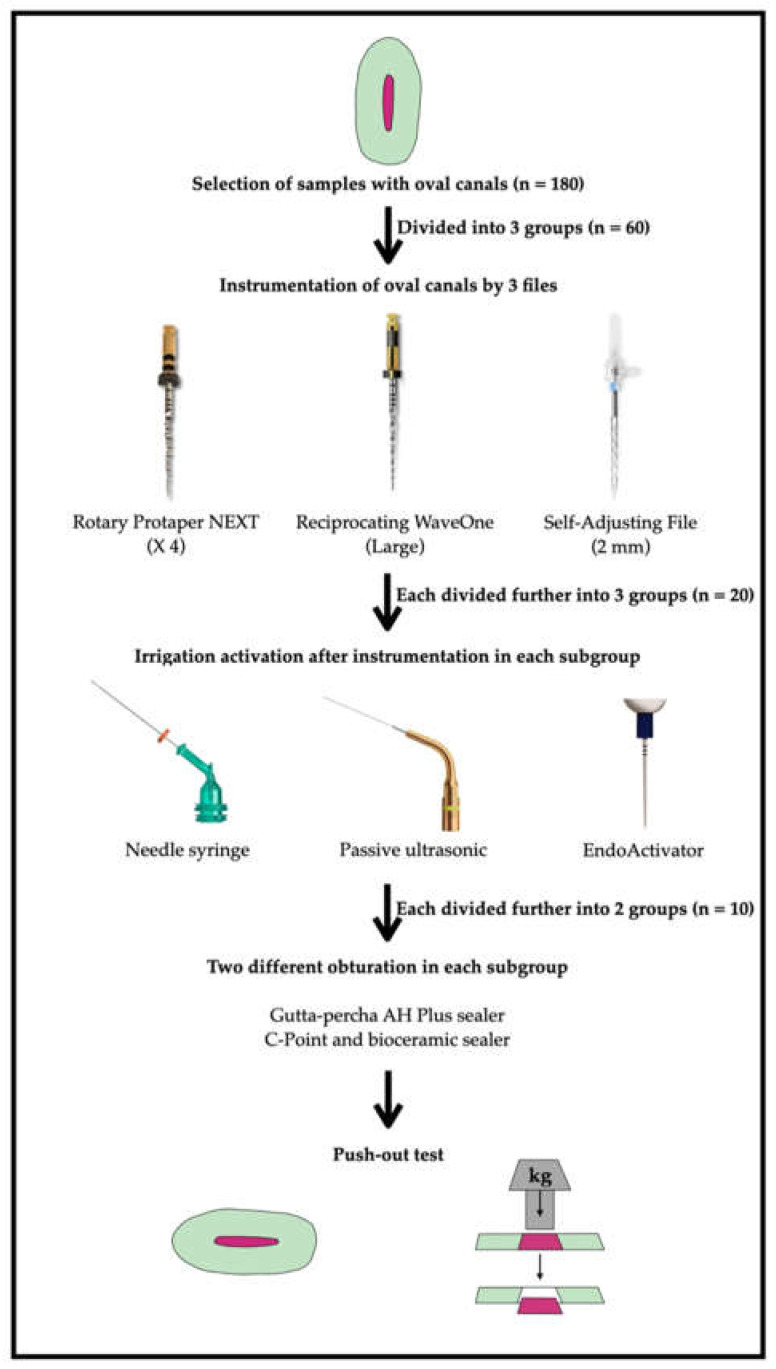
Flowchart of the experimental design.

Treatment protocols in all groups consisted of 3 stages: (i) canal instrumentation using various file systems including irrigation, as required by each file manufacturer, (ii) final irrigation with or without activation of the irrigant and (iii) obturation of the canals using one of the two root filling methods (Figure 1).

### 2.3. Root Canal Instrumentation

The working length of the specimens was established using a size 10 K-file (Mani. Tochigi, Japan). Hand K-files (Mani) up to size 25, using 5.25% sodium hypochlorite (NaOCl) as the irrigant, were used for the achievement of the initial glidepath for all root canals. The specimens were then randomly divided into 3 groups based on the intended instrumentation protocol (*n* = 60): group 1: ProTaper NEXT (PTN, Dentsply Maillefer, Ballaigues, Switzerland), up to X4 size; group 2: WaveOne “large” file (WO, Dentsply Maillefer); group 3: Self-adjusting File, 2 mm diameter (SAF, ReDent, Raanana, Israel).

The instruments were used according to the manufacturers’ instructions, including irrigation protocols during instrumentation, as recommended by each of the manufacturers. During these procedures, 5.25% NaOCl was used as the irrigant. Irrigation during canal instrumentation in the ProTaper NEXT and WaveOne groups was performed using a syringe and a 31 G side-port needle (Ultradent, South Jordan, UT, USA). In the SAF group, irrigation was performed through the hollow file using a VATEA irrigation pump (ReDent), which is an integral part of the SAF system. The volume of irrigating solution and irrigation time during canal instrumentation were standardized to a total of 16 mL and 4 min, respectively, for all instrumentation methods.

### 2.4. Final Irrigation Activation

After canal preparation was completed, the final irrigation was applied with or without activation of the irrigant. The specimens of each group were divided into three subgroups based on the final irrigant delivery/activation protocol (*n* = 20): subgroup A: conventional syringe and needle irrigation (SN) using a 31 G side-port needle (Ultradent), with no additional activation, which served as a negative control; subgroup B: passive ultrasonic irrigation (PUI, IrriSafe, Satelec Acteon, Merignac, France); subgroup C: sonic activation (EndoActivator, Dentsply Tulsa, Tulsa, OK, USA). For subgroups B and C, the irrigant was activated for 30 s, following which fresh irrigant was placed into the canal. This process of activation and replacement of the irrigant was repeated until a total irrigation time of 4 min was reached (8 cycles of 30 s each). The total volume of irrigant at this stage was standardized to 8 mL in all groups.

Following the completion of the final irrigation/activation, the canals were rinsed with 2 mL distilled water and irrigated with 2 mL 17% EDTA for 2 min to remove the smear layer. The canals were then rinsed with 5 mL distilled water and dried using paper points (Dentsply Maillefer).

The samples of each group were then obturated with one of the following methods (*n* = 10): gutta-percha with AH Plus using the lateral compaction (GP-AH), or C-Point with EndoSequence BC sealer (C-EBC), which was performed according to the C-Point manufacturer’s instructions [6]. The samples were then stored at 37 °C and 100% moisture for 7 days to permit total setting of the sealers.

### 2.5. Measurement of Dislocation Resistance by Push-Out Bond Strength Test

The dislocation resistance of the root fillings was determined using the micro-push-out bond strength test (Figure 1) [6,12]. Each sample was submerged in an epoxy resin placed in a custom-built split-ring copper mold. Following the setting of the resin, a 1 mm slice was cut horizontally at 5 mm from the root tip (including a slice 5–6 mm from the apex), using a high-precision saw (Buehler, Lake Bluff, NY, USA) in conjunction with a diamond-smeared disk (Swiss NF Metals, Markham, ON, Canada). Water cooling was used for the specimens filled with gutta-percha and AH-Plus, while no water was used for the C-EBC samples (as the C-Points are hydrophilic in nature and expand when exposed to moisture). A digital caliper (Avenger Products, North Plains, OR, USA) was used to measure the thickness of each sample, and the coronal surface of each slice was marked with an indelible marker.

The root fillings of these slices were then subjected to a compressive load using a universal testing machine (Zwick, Memmigen, Germany) at a crosshead speed of 0.5 mm/min. A barrel-shaped stainless-steel plunger of either 0.5 mm or 0.3 mm diameter (as required by an individual case) was used, which was positioned to touch only the root filling materials. A push-out force was devoted in an apicocoronal direction until root fillings were dislodged suggesting bond failure (Figure 1); bond failure was manifested by extrusion of the filling material and by a sudden drop in load deflection. The force that caused bond failure was recorded in newtons (N), and the push-out bond strength was calculated and expressed in megapascals (MPa) using the formula of force divided by the adhesion surface area. The adhesion area was determined as reported previously [13,14].

The canal surface of the samples was further viewed under a stereomicroscope at 25× magnification after the push-out test to determine the failure mode. The samples were marked with methylene blue dye to help distinguish between dentine surfaces coated in sealer (cohesive failure) and dentine surfaces without sealer (adhesive failure) [6].

### 2.6. Data Presentation and Analysis

The main outcome variable in this study was dislocation resistance in MPa. Data analysis by the Shapiro–Wilk test showed normal distribution of the data; thus, the utilization of parametric statistical tests was justified. A three-way analysis of variance (ANOVA) was performed considering the instrumentation protocol, the root filling material and the final irrigant activation protocol as 3 independent variables and push-out bond strength (dependent variable) as the outcome. A one-way ANOVA was employed to identify the effect of each of the independent variables on the outcome. The alpha-type error was set at 0.05 for all statistical analyses (Statistical Package for Social Sciences (SPSS) software for MacBook version 20).

## 3. Results

The mean push-out bond strength values appear in Table 1. The analysis of the data by the three-way ANOVA manifested that the instrumentation protocol and type of root filling had a significant impact on the dislocation resistance of both filling materials (*p* < 0.05), while the bond strength was unaffected by the irrigant activation technique (*p* > 0.05) (Table 1).

### 3.1. Effect of Instrumentation Protocol

Specimens that were subjected to the PTN or WO instrumentation showed lower bond strength values than the values of those subjected to the SAF system (Group 3), which served as a positive control. However, these differences were statistically significant only when the roots were filled with C-EBC (*p* < 0.05) (Table 1).

For each of the root filling materials, there was no significant difference between either of the two activation protocols and delivery of the final irrigant with a syringe and needle without activation, which served as a negative control (*p* > 0.05). Preparation of the canals with WO exhibited a higher bond strength than that with PTN for both root filling materials (irrespective of the irrigant activation protocol), but these values were significantly different for only the roots filled with C-EBC (*p* < 0.05).

### 3.2. Effect of Final Irrigant Activation Protocol

The bond strengths exhibited by either of the two activation protocols of the final irrigant, compared with the final irrigation that was done with a syringe and needle, with no activation, which served as negative control, did not differ (*p* > 0.05). While PUI exhibited marginally higher (or similar) bond strength values for the two root filling materials, irrespective of the instrumentation protocol, the differences between these values were not significant (*p* > 0.05).

### 3.3. Effect of Root Filling Material

C-EBC showed higher mean bond strength values than those of the gutta-percha with AH Plus. However, these values were significantly higher in only the subgroups that underwent endodontic procedure with SAF (*p* < 0.05).

When the root canals underwent procedure with SAF and final irrigant activation was performed with PUI, the C-EBC root fillings demonstrated significantly high bond strength values (Table 1). However, this bond strength was not significantly different from the bond strength of C-EBC in the canals in which SAF was used and final irrigant was delivered/activated with a syringe and needle or sonic activation (*p* > 0.05) (Table 1).

### 3.4. Mode of Failure

The modes of failure (adhesive, cohesive, mixed) in the specimens are presented in Table 2. In specimens that were instrumented with PTN or WO irrespective of the irrigation regime or the obturating material, adhesive failure was significantly pronounced (*p* < 0.001), compared to the specimens that were subjected to SAF instrumentation. This parameter did not differ between the PTN and WO groups (*p* > 0.05). In the group in which canals were instrumented with the SAF and filled with the C-EBC sealer, no case of pure adhesive failure was detected in any of the parts of the canal.

## 4. Discussion

The present investigation revealed that activation of the final irrigant with either sonic or ultrasonic devices had no notable effect on the bond strength of the root filling materials in oval canals, as compared to non-activated final irrigation. Hence, the basic hypothesis of this study was rejected. It seems that the expectation that when activated, the irrigant may effectively clean the debris that were left in or packed into canal recesses by rotary or reciprocating files in oval canals was not fulfilled. These findings are in agreement with previous literature [11]. The present findings are also in accordance with the concept presented by Metzger et al. [15] that effective cleaning of oval canals requires mechanical debridement of the recesses left after instrumentation of oval canals with rotary or reciprocating files.

Root canal dentin wall conditioning has been shown to possess a significant impact on the dislocation resistance of root filling materials [16]. The results of a push-out strength test may be affected by the adherence of the root filling to the canal walls and may thus represent an inherent material property of the root filling system used. Nevertheless, push-out strength also may represent the cleaning efficacy of root canal instrumentation systems or final irrigation methods; this may be true particularly in the case of oval root canals. Intimate contact between the root canal filling substance and all of the root dentin surface, may also potentially affect the push-out strength of root fillings [6,17]. The aforementioned is especially true with oval root canals, in which there is difficulty in effectively cleaning the buccal and/or lingual canal recesses [3,15].

While several studies have assessed the bond strength of gutta-percha + AH Plus [16,18], there is minimal literature on the influence of the instrumentation system used [6] but none on the method of final irrigant activation on the bond strength of root canal fillings made of C-Point with the EndoSequence BC sealer.

The present study also included one of the most widely used rotary files: ProTaperNext (Dentsply Maillefer, Ballaigues, Switzerland). The assumption was that rotary files may have a lesser tendency to pack debris into canal recesses, and thus may result in a higher push-out bond strength than the reciprocating files. The choice of instruments was based on the contemporary controversy in endodontics related to the kinematics of the instruments, the rotation, reciprocation, or trans-line vibratory method. The primary difference between the three file systems used in the present study is their ability to clean recesses of oval canals or, alternatively, to actively pack debris into these recesses [3,4,6,11,15], as well as their ability or inability to effectively contact and remove dentin from all the walls of oval root canal systems. Debris that is left in or pushed into unshaped buccal or lingual ambush of an oval root canal may impede intimate adaptation of the root filling to all surfaces of root canal dentin, thereby reducing the adhesion [6].

The results of the present study show that the bond strength of tested root filling materials in canals shaped with rotary or reciprocating files was lower than that registered in canals treated with the SAF, which served as a positive control. This result is in agreement with previously published data [6].

The SAF system is essentially a cleaning shaping irrigation system and implements a unique action. The cleaning ability of SAF in oval root canals, compared to that of rotary files in oval root canals, was previously reported [2,4,11].

The improved bond strength of root filling materials in oval canals instrumented with the SAF system may have resulted from a lack of uninstrumented areas. Two recent investigations revealed the inability of PTN and WO to adhere to the surfaces of canal walls of mandibular molars. The uninstrumented area associated with these files were almost 41% for PTN and almost 29% for WO [1,19]. This could explain the results obtained in the current investigation with the samples instrumented with PTN and WO exhibiting lower values of dislocation resistance of the root canal fillings.

Moreover, the SAF lacked areas where debris was left in or vigorously packed into recesses of the canal. The presence of such debris in oval canals instrumented with either rotary or reciprocating files was well-documented by DeDeus et al. [4,11,12] and more recently by Martins et al. [19]. The presence of such debris most likely reduces the area of the root canal wall that comes into contact with the root canal filling compared with cleaner recesses that result from the SAF instrumentation [12]. This may have resulted in the higher bond strength that was previously found in the oval canals treated with the SAF system [6].

The push-out bond strength is a well-acknowledged method for determining the adhesion of root filling materials [20]. Some authors recommend that the ideal adhesion should be assessed when the root canals are filled with only the sealer and no core material [21]. Other authors suggest simulating the clinical situation of filling root canals with the core material and sealer [6,12,16]. Indeed, it is possible to obliterate root canals with the sealer alone, but retreatment is considered difficult because sealers such as AH Plus or BC sealers may set with a very hard consistency [13]. Furthermore, the C-Point with the EndoSequence BC sealer may be considered as an “obturation system” in which each of its elements is an essential component [6]. Hence, in the present study, root canals were filled with the core materials and sealers instead of the sealer alone.

The method of root filling material adhesion to dentin is of considerable interest for understanding these results. It is unknown if sealer penetration has a significant effect on bond strength [1,4,6]. It was beyond the scope of the present work to evaluate this parameter. The adhesion of the root filling material to dentin may be affected by the irrigants used and thus affect the push-out bond strength. Recent data demonstrate that the epoxy resin sealer AH Plus covalently bonds to the organic phase of dentin [22]. It has also been demonstrated that a final rinse with NaOCl reduces the bond strength of AH Plus [16,23,24]. Contrarily, there is also evidence showing that a final flush with NaOCl or EDTA reduces the dislocation resistance of tricalcium silicate-based sealers such as EndoSequence BC. To counteract the effects of these chemically active irrigants on the adhesion of the root filling materials, a final rinse with distilled water was used in the present study in all groups to remove all possible traces of the irrigants.

The hydrophilic peculiarity of the EndoSequence bioceramic sealer may have prospectively resulted in an increased intimate contact with the canal walls compared to that of the hydrophobic AH Plus sealer [13]. When laid bare to moisture, the outer layer of the C-Point demonstrates a non-isotropic spiral expansion pushing the BC sealer radially, enabling it to transform to irregular spaces [6]. Furthermore, the BC sealer used with C-Point is a slow setting hydrophilic sealer that, reportedly, has good adaptation to radicular dentin [12,14]. The relaxed setting of this sealer (4–10 h) possibly allowed the inflating C-Point to thrust the sealer into the dentinal tubules, in addition to obtaining as proximate a contact as possible with the canal walls [12,14]. Furthermore, one may assume that the unhurried setting of the BC sealer combined with the slow inflation of the C-Point when exposed to moisture may have possibly plunged the sealer into places where lateral compaction with the AH-Plus sealer could not reach.

In terms of failure modes (cohesive, mixed, and adhesive), the mixed and cohesive types are perhaps the most common for the AH Plus [25], while the cohesive type was more frequent for the BC Sealer, indicating the cements’ high adherence to the dentinal wall [25]. In the current study, contrasting results were observed with respect to gutta-percha and AH Plus group. For GP-AH fillings in samples instrumented by PTN and WO the adhesive failure was more pronounced regardless of the irrigation protocol. These findings support the notion that the files invariably form debris that may be packed in the canal recesses, especially in oval canals [4,6]. The different irrigation protocols also failed to influence the dislocation resistance of the root fillings which has been reported in the literature [3]. The results of the type of bond failure between the root filling material and the dentin of the canal wall may be of interest, expanding the horizon of the drawbacks of solid cored rotary/motorized files when used for instrumenting oval canals.

Recently, other new types of instruments were introduced, which were specially designed to address the shaping and cleaning of oval canals, such as the XP-endo shaper and XP-endo finisher [26,27,28,29,30]. It will be of interest to test the effect of these instruments on the bond strength of root canal fillings in oval canals, nevertheless, such comparison was beyond the scope of the present study.

The present investigation seemed to have limitations. First, the analysis was limited to oval canals. Second, the current study did not investigate the influence of different irrigation activations on the potential of debris to be removed from the radicular dentin surface. Furthermore, the current research was conducted on a single level (5 mm coronally from the root apex where the root canal exhibits a pronounced oval canal at this level). However, measuring dislocation resistance in the apical third is one of the areas that might be researched further. Future research should investigate these constraints more.

## 5. Conclusions

The dislocation resistance of root fillings in oval canals prepared with either rotary or reciprocating files was lower than that prepared with the SAF, which served as a positive control. Irrigant activation with either sonic or ultrasonic devices had no significant effect, compared to irrigation with no activation. Adhesive failure was significantly more, irrespective of the irrigation protocol or the obturation material used in specimens instrumented with PTN and WO. The C-Point accompanied by the EndoSequence BC sealer produced significantly higher push-out bond strength values than those of the gutta-percha in conjunction with AH Plus.

## Figures and Tables

**Table 1:** Push-out bond strength of root fillings made of two root-filling materials, in oval root canals prepared with three different instrumentation methods and subjected to different activation protocols of the final irrigant (*n* = 10).

Method	SN		PUI		SA	
	GP-AH	C-EBC	GP-AH	C-EBC	GP-AH	C-EBC
PTN	1.4 ± 0.3 † a, A	1.6 ± 0.3 a, A	1.7 ± 0.3 a, A	2.0 ± 0.6 a, A	1.6± 0.3 a, A	1.9 ± 0.4 a, A
WO	1.8 ± 0.3 a, A	3.1 ± 0.4 b, B	2.0 ± 0.3 a, A	3.3 ± 0.3 b, B	1.9 ± 0.2 a, A	3.1 ± 0.4 b, B
SAF	2.9 ± 0.5 b, A	**4.6 ± 0.5** c, B	3.0 ± 0.7 b, A	**4.9 ± 0.3** c, B	2.5 ± 0.3 b, A	**4.2 ± 0.5** c, B

† MPa, means ± standard deviations. PTN: ProTaper Next. WO: WaveOne. SAF: Self-adjusting File (positive control for instrumentation methods). SN: syringe and needle irrigation (negative control for irrigant activation methods). PUI: passive ultrasonic irrigation. SA: sonic Activation. GP-AH: gutta-percha + AH Plus. C-EBC: C-Point + Endosequence BC sealer. Methods of instrumentation are compared in the vertical columns. Mean values that share a lowercase script letter (along each column) were not significantly different, while those with different letters were different at the *p* < 0.05 level. Methods of activation of the final irrigant are compared along the horizontal rows. Mean values that share an uppercase script letter (along horizontal rows) were not significantly different, while those with different letters were different at the level of *p* < 0.05. The highest bond strength values have been bolded and italicized. Since there was no significant difference between the mean bond strength values of each material for the different irrigant activation protocols, they have not been represented in the table.

**Table 2:** Modes of failure in percentage and number of samples (n) after push-out bond strength test of root fillings.

Method	PTN						Total Specimens
	SN		PUI		SA		
	GP-AH	C-EBC	GP-AH	C-EBC	GP-AH	C-EBC	
Adhesive	70 (7)	60 (6)	50 (5)	40 (4)	50 (5)	40 (4)	60
Cohesive	10 (1)	30 (3)	20 (2)	30 (3)	30 (3)	20 (2)	
Mixed	20 (2)	10 (1)	30 (3)	30 (3)	20 (2)	40 (4)	
	WO						
Adhesive	60 (6)	60 (6)	60 (6)	50 (5)	50 (5)	40 (4)	60
Cohesive	20 (20)	10 (1)	30 (3)	20 (2)	30 (3)	30 (3)	
Mixed	20 (20)	30 (3)	10 (1)	30 (3)	20 (2)	30 (3)	
	SAF						
Adhesive	10 (1)	00 (0)	00 (0)	00 (0)	20 (2)	00 (0)	60
Cohesive	70 (7)	30 (3)	60 (6)	20 (2)	70 (7)	30 (4)	
Mixed	20 (2)	70 (7)	40 (4)	80 (8)	10 (1)	70 (7)	

## Data Availability

All the data related to the study is presented in the manuscript.
